# Immune-related adverse events with severe pain and ureteral expansion as the main manifestations: a case report of tislelizumab-induced ureteritis/cystitis and review of the literature

**DOI:** 10.3389/fimmu.2023.1226993

**Published:** 2023-10-06

**Authors:** Qihao Zhou, Zhiquan Qin, Peiyuan Yan, Qunjiang Wang, Jing Qu, Yun Chen

**Affiliations:** Cancer Center, Department of Medical Oncology, Zhejiang Provincial People’s Hospital, Affiliated People’s Hospital, Hangzhou Medical College, Hangzhou, China

**Keywords:** immune-related adverse events, tislelizumab, cystitis, ureteritis, case report

## Abstract

Immune checkpoint inhibitor (ICI) is an up-to-date therapy for cancer with a promising efficacy, but it may cause unique immune-related adverse events (irAEs). Although irAEs could affect any organ, irAEs-induced whole urinary tract expansion was rarely reported. Herein, we reported a 27-year-old male patient with thymic carcinoma who received the treatment of tislelizumab, paclitaxel albumin and carboplatin. He was hospitalized for severe bellyache and lumbago after 6 courses of treatment. Antibiotic and antispasmodic treatment did not relieve his symptoms. The imaging examinations reported whole urinary tract expansion and cystitis. Therefore, we proposed that the patient’s pain was caused by tislelizumab-induced ureteritis/cystitis. After the discontinuation of tislelizumab and the administration of methylprednisolone, his symptoms were markedly alleviated. Herein, we reported a rare case of ICI-induced ureteritis/cystitis in the treatment of thymic cancer and reviewed other cases of immunotherapy-related cystitis and tislelizumab-related adverse events, which will provide a reference for the diagnosis and treatment of ICI-related irAEs.

## Introduction

Immune checkpoint inhibitor (ICI) is an emerging immunotherapy for cancers. However, since ICI will activate immune responses, it may cause unique immune-related adverse events (irAEs). irAEs can affect different organs and reduce the survival benefit of immunotherapy if untreated (1). In some cases, irAEs will endanger the lives of patients (2, 3).

Tislelizumab (BGB-A317) is a humanized anti-programmed death receptor 1 (PD-1) monoclonal antibody. In clinical studies, tislelizumab has shown promising anti-tumor activity in various solid tumors (4). In these studies, tislelizumab-related adverse events are briefly recorded (5), but few adverse events related to the urinary system are reported. In other case reports, tislelizumab is suggested to induce various immune-related adverse events (2, 3, 6–15). A previous case report indicated that tislizumab could induce ureteritis and cystitis in patients with esophageal cancer (15). However, the chief complaint of the patient in that case report differed from that in our case. Moreover, another PD-1 inhibitor, sintilimab, was reported to cause cystitis and ureteritis (16). Our patient was hospitalized for bellyache with paroxysmal lumbago. He had no obvious symptom of frequent urination, urgency, and pain in urination. The positron emission tomography/computed tomography (PET/CT) and magnetic resonance urography (MRU) scans revealed an expanded whole urinary tract, which is rarely reported. Therefore, this is the first report of ICI-induced ureteritis and cystitis during the treatment of thymic cancer. In addition, we reviewed several cases of immune-induced cystitis (15–25), in which the main manifestations of patients were frequent urination, dysuria, pain on urination, nocturia or incontinence. Hence, our report would provide a reference for the diagnosis and treatment of patients who received ICI and complained of bellyache.

## Case presentation

**Figure 1:**
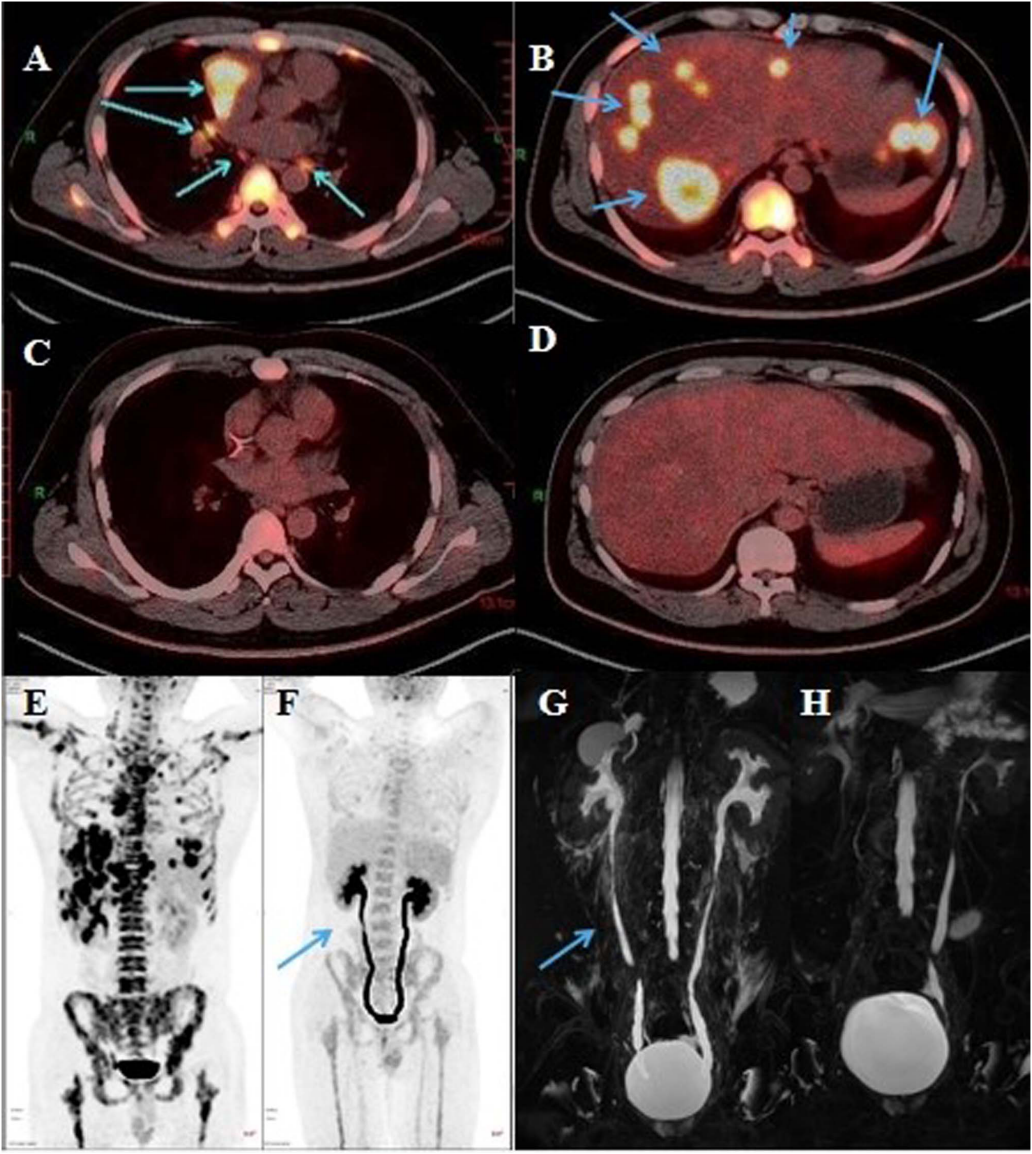
Representative radiological images of the patient. **(A)** Mediastinum tumor and **(B)** hepatic metastatic lesion on PET/CT (April 11, 2022). **(C)** Mediastinum tumor and **(D)** hepatic metastatic lesion on PET/CT (August 24, 2022). **(E)** PET/CT (April 11, 2022). **(F)** Bilateral ureters were slightly dilated on PET/CT (blue arrow, August 24, 2022). **(G)** Bilateral ureteral wall was slightly thickened and whole ureteral was full expanded on urinary MRU (blue arrow, August 31, 2022. **(H)** Right ureter expansion was improved, and ureteral exudation was absorbed on urinary MRU (September 22, 2022).

A 27-year-old male patient was admitted to Zhejiang Hospital due to chest pain. He had a history of fatty liver and kidney stones with no history of smoking and drinking. He did not have a medical history of hypertension, diabetes, kidney disease, or hepatitis. His father had hepatitis B, and no family members had a tumor history. The CT showed anterior superior mediastinal and liver mass on March 30, 2022. Pathological results of liver puncture indicated poorly differentiated carcinoma with necrosis. Then the patient was admitted to our hospital on April 9, 2022. The immunohistochemical examination of the liver mass indicated CK (Pan) (+), CD5 (+), CgA (-), SYN (-), P63 (scattered cells+), CD117 (+), GLUT-1 (+), Muc-1 (+), CD3 (-), P40 (scattered cells+), CD56 (-), CK20 (-), Ki67(+≈90%) (**Appendix Figure S1**), which suggested that it was liver metastasis of thymic carcinoma. The expression of PD-L1 is positive in 70% of tumor cells (clone 22C3, Dako, Glostrup, Denmark). Also, we sequenced the 520 pan-cancer genes in formalin fixation and paraffin embedding specimens (**Appendix Table S1**). The tumor mutation burden (TMB) was 10.2 Muts/Mb, which was higher than 99% thymic carcinoma. The ratio of mutation at microsatellite site was 1.65% (2/121), which indicated microsatellite stability (MSS). PET/CT showed that the size of the tumor in anterior superior mediastinum was 4.1 cm × 3.5 cm, and standard uptake value (SUV) max was 16.6. The boundaries between the tumor and adjacent superior vena cava, pericardium and mediastinal pleura were not clear (**Figure 1A**). The tumor had liver (**Figure 1B**), lymph nodes and bone metastasis. Based on these results, the tumor was staged as pTxN1M1. His performance score (PS) was 1 (PS ranged from 0 to 6, and the lower value indicated better physical condition). The patient began to receive chemo-immunotherapy on April 14, 2022. He was administered with paclitaxel albumin (CSPC, OUYI, Pharmaceutical Co, Ltd) (200 mg at Day 1, Day 8), carboplatin (Bristol-Myers Squibb S.r.l., 0.3 g at Day 1, Day 8) and tislelizumab (Baize’an, BeiGene Ltd., Beijing, China, 200mg at Day 1) for 6 courses. Chest CT and liver MRI showed significant reduction of the tumor and the treatment reached partial response (PR). On August 19, the patient developed bellyache, which was day 7 since the last treatment course. The bellyache last for 2 days, with significant pain on the left side and paroxysmal lumbago and no gross hematuria. The symptom could relieve on itself.

**Figure 2:**
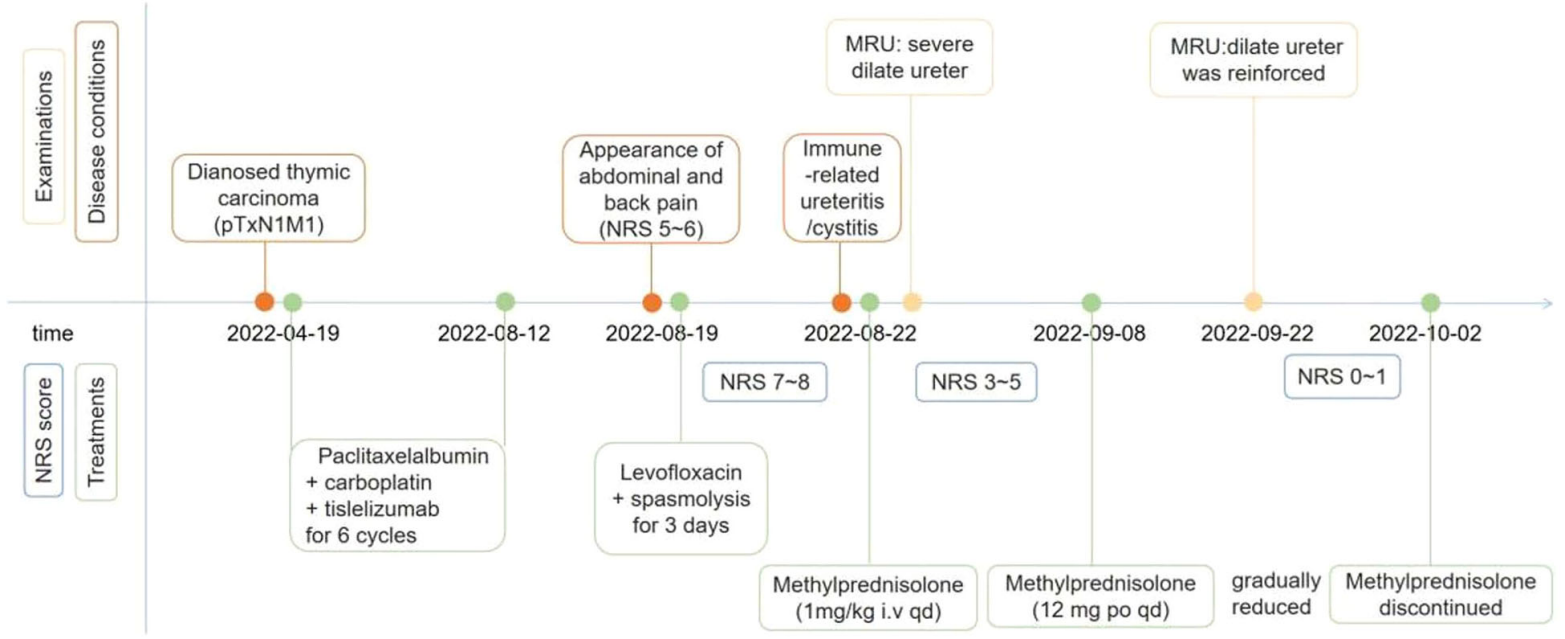
The timeline of the patient’s symptom, diagnosis and treatments.

The patient had percussion pain (+) in renal area. Urinalysis showed red blood cells (+++) and white blood cells (++). His white blood cells in routine blood examination was 16.76 (normal 3.5-9.5)×10^9/L. B-ultrasound examination in bladder was normal and that in abdomen showed bilateral kidney stones. The level of glutamic pyruvic transaminase (GPT), serum amylase and serum creatinine was normal. Therefore, we proposed that the patient had a urinary tract infection due to the urinary calculi, and we administered levofloxacin (0.5 g) and phloroglucinol (80 mg) for 3 days. However, his pain did not subside. Initially, the patient had a breakthrough pain once a day, with the Numerical Rating Scale (NRS) score of 7-8. Later, the frequency of pain increased to 3 times a day and the pain was obvious when urinating, based on which we conducted urine culture, but failed to identify any pathogen. PET/CT scan showed that the mass at the right anterior mediastinal was smaller than before (2.3 cm × 2.2 cm vs 4.1 cm × 3.5 cm), and the metabolism was reduced (SUVmax 4.5 vs 16.6). There was no significant increase of 18F-Fluorodeoxyglucose (FDG) fluorodeoxyglucose (FDG) metabolism in metastatic tumor at lymph nodes and liver (**Figures 1C, D**). Meanwhile, PET/CT revealed, compared to the previous images (**Figure 1E**), poor bladder filling, slightly thickened bladder wall, slightly enlarged left kidney, increased FDG metabolism in bilateral renal parenchyma, dilated bilateral ureters with smooth excretion, and no obvious ureteral calculus (**Figure 1F**). Consistent with previous findings, MRU scan showed that bilateral ureteral wall was slightly thickened and whole ureteral fully expanded (**Figure 1G**). Cystoscopy indicated cystitis (**Appendix Figure S2**). Based on these results, a multi-disciplinary treatment (MDT) meeting was organized. The cause of pain excluded the urinary infection, tumor metastasis and nephrolithiasis, and the pain was most probably caused by tislelizumab-induced ureteritis/cystitis. The patient was administered with methylprednisolone intravenously, 1 mg/kg once daily and tislelizumab was discontinued. The patient’s persistent bellyache and lumbago were basically relieved, the intensity of paroxysmal pain reduced, and the pain after urination improved after 3 days of treatment. After he was discharged on September 8, Methylprednisolone (12 mg) was administered orally once daily. During the follow-up, the patient had slightly poor sleep but had no obvious adverse events such as drug allergy, gastric bleeding, and edema, etc. The patient’s pain did not recur and the dose of methylprednisolone was decreased gradually. On September 22, the MRU indicated that the dilation of left kidney and right ureter was improved compared to those on August 31, and the around ureteral exudation was absorbed. (**Figure 1H**). Methylprednisolone was finally discontinued about 40 days after treatment. Because the patient’s pain was caused by ICI rather than chemotherapy drugs, the patient received paclitaxel albumin and platinum again on September 22, 2022. No similar pain or urinary symptoms occurred during the follow-up. The timeline of treatment course was summarized in **Figure 2**.

## Discussion and review of the literature

The structure of tislelizumab has been modified to maximally block the binding of PD-1 to programmed death ligand 1 (PD-L1) (4). The binding of Fcγ receptors (FcγRs) will impair the anti-tumor activity of anti-PD-1 antibody (26). Several Fc-hinge regions of tislelizumab has been muted to minimize its binding to FcγRs. Up to now, tislelizumab has been approved for the treatment of various tumors, including classical Hodgkin’s lymphoma, urothelium cancer, lung adenocarcinoma, non-squamous cancer, liver cancer, esophageal squamous cancer, nasopharyngeal cancer, advanced colorectal cancer, and solid tumors with microsatellite instability-high (MSI-H) or mismatch repair protein deficiency (dMMR) MSI-H or dMMR in China. However, adverse events may occur during tislelizumab treatment. Existing clinical studies suggested that adverse effects of tislelizumab included anemia, leukopenia, thrombocytopenia, nausea, increased aspartate transaminase (AST), neutropenia, fatigue, decreased appetite, vomiting, musculoskeletal pain, constipation, hypoproteinemia and rash (4, 5). To better understand the adverse events of tislelizumab, we searched for available case reports and reviewed tislelizumab-associated adverse events (**Table 1**). Although irAEs can affect any organ, tislelizumab-related irAEs in the urinary system are rarely reported. A previous case report indicated that tislizumab could induce ureteritis and cystitis in patients with esophageal cancer (15). However, the chief complaint of the patient in that case report differed from that in our case.

**Table 1:** Tislelizumab-related adverse events in available case reports.

Malignancies	Age(yr)	Gender	Diagnosis	Cycles	Reference
NSCLC	75	F	Lichen planus pemphigoides	1	Kerkemeyer et al. (6)
NSCLC	73	M	Colitis with Clostridium difficile positive	≈8	Ni et al. (7)
NSCLC	74	M	Nephrotic syndrome, membranous nephropathy	11	Chen et al. (8)
NSCLC	71	M	Severe myositis, myocardial damage, hepatic damage, secondary adrenal insufficiency	1	Deng et al. (3)
AGC	66	F	Pulmonary embolism, deep vein thrombosis	1	Fu et al. (2)
UUC	66	M	Myocarditis	UN	Hu et al. (9)
NSCLC	56	M	Herpetiform pemphigus	6	Zhang et al. (10)
AGC	58	M	Hypothyroidism and adrenal insufficiency	6	Baek et al. (11)
AGC	59	M	Subclinical hypothyroidism and adrenal insufficiency	13	Baek et al. (11)
CHL	25	F	Grade 2 immune-related pneumonitis	2	Zhou et al. (12)
LACRC	65	M	Severe myasthenia gravis, myocarditis, and rhabdomyolysis	1	Wang et al. (13)
CHL	26	F	Tumor flare reaction	4	Zhu et al. (14)
EC	49	F	Ureteritis and cystitis	6	Li et al. (15)
TC	27	M	Ureteritis/cystitis	6	This case

NSCLC, non-small cell lung cancer; UUC, ureteral urothelial cancer; AGC, advanced gastric cancer; LACRC, locally advanced colorectal cancer; CHL, classic Hodgkin lymphoma; EC, esophagus cancer; TC, Thymic carcinoma; UN, unknown; M, male; F, female; mPSL, methylprednisolone; PSL, prednisolone; ≈, about.

Thymic carcinomas are rare malignancies. For unresectable or metastatic thymic carcinomas, chemotherapy is the standard treatment. ICIs are new drugs with promising efficacies in cancers. A phase 2 clinical trial of pembrolizumab (27), an anti-PD-1 antibody, IN 40 patients with thymic carcinoma showed that the overall response rate (ORR) was 22.5% and the median progression-free survival (mPFS) was 4.2 months. Moreover, those with high PD-L1 expression benefit more from pembrolizumab treatment. In our case, PD-L1 was 70% positive in patient’s tumor cells with a high TMB. A study showed that patients with high TMB and PD-L1 expression had a high rate of durable clinical benefit from ICIs treatment (28). Therefore, our patient is likely to benefit from ICI. However, due to the financial issue, the patient chose another ICI, tislelizumab, for subsequent treatment.

Here, we report a case of tislelizumab-induced ureteritis and cystitis. Up until now, there is no standard for the diagnosis of immune-related cystitis, where cystoscopic biopsy may help. In our case, we suspected that patient’s bellyache was caused by kidney stones since abdominal B-ultrasound showed bilateral kidney stones. However, no stone was found on urinary CT, which might be due to the small size of the stone that was not shown on CT. Since small kidney stones rarely caused such severe and long-term pain, we further performed PET/CT and found no tumor metastasis of the urinary system. Nevertheless, ureteral expansion was identified by PET/CT and MRU, and cystoscopy suggested cystitis. After excluding kidney stones, tumor invasion, and urinary tract infection, we considered that the patient’s pain was caused by immune-related ureteritis and cystitis. After the steroid administration, the pain was markedly alleviated, and the following MRU suggested that ureteral expansion was relieved. Therefore, we confirmed the diagnosis that the patient’s pain and ureteral dilatation were caused by tislelizumab-induced ureteritis and cystitis.

irAEs are toxicities caused by non-specific activation of the immune system and can affect almost any organ (29). However, the exact mechanism of irAEs is not clear, which may involve the activation of various inflammatory cells, such as Th17 and other types of cells (29). Other studies indicated that irAEs might occur because of impaired immune tolerance and molecular mimicry (21). Studies had found that PD-L1 was expressed in bladder tissue in patients with severe bladder inflammation. Therefore, it is speculated that PD-1/PD-L1 mAb-induced cytotoxic T-cell activation may simultaneously target at cancer and normal urothelial cells (15). A meta-analysis showed that the incidence of irAEs significantly increased when ICI was combined with chemotherapy (21).

In published clinical trials and case reports, there is rarely report on tislelizumab-induced ureteritis and cystitis. Therefore, we referred to previous case reports on autoimmune cystitis caused by more than tislelizumab (**Table 2**). Among these cases, 50% (7/14) patients received nivolumab, 14% (2/14) patients received pembrolizumab, 21% (3/14) patients received sintilimab, 7% (1/14) patient received atezolizumab, and 7% (1/14) patient took tislelizumab. Moreover, there is no clear timing for the onset of immune-related cystitis during ICIs treatment. The main manifestations of patients in these case reports were frequent urination, dysuria, urination pain, nocturia, incontinence, or diarrhea. Only two of them had low back pain, and one received tislelizuma (16) and another one received sintilimab (15). As a comparison, the manifestation of our patient was bellyache, which was different from that in these immune-induced cystitis cases. More importantly, the ureter of this patient was expanded. Such cases with irAEs in urinary system and expanded ureter are rarely reported. A case of irAEs induced by tislelizumab exhibited different manifestation. Therefore, this is the first report of autoimmune ureteritis/cystitis in the treatment of thymic cancer. Our case will provide a reference for the diagnosis and treatment of ICI-induced ureteritis and cystitis manifested by bellyache.

**Table 2:** Clinical information of the case reports of irAEs cystitis.

Malignancies	Age(yr)/sex	Gender	ICIs	Cycles of onset from ICIs	Reference
NSCLC	62	M	Nivolumab	3	Ozaki et al. (17)
NSCLC	50	M	Nivolumab	7	Shimatani et al. (18)
NSCLC	60	M	Nivolumab	12	Shimatani et al. (18)
NSCLC	78	F	Pembrolizumab	6	Ueki et al. (19)
NSCLC	47	M	Nivolumab	18	Yajima et al. (20)
SCLC	51	M	Nivolumab	5	Zhu et al. (21)
NSCLC	53	M	Sintilimab	3	Tu et al. (16)
GC	56	M	Sintilimab	5	Wang et al. (22)
BC	67	F	Atezolizumab	4	Obayashi et al. (23)
NSCLC	56	M	Pembrolizumab	6	He et al. (24)
Primary lung cancer	60	M	Nivolumab	77	Fukunaga et al. (25)
EC	49	F	Tislelizumab	6	Li et al. (15)
GC	62	F	Sintilimab	3	Li et al. (15)
GC	49	F	Nivolumab	2	Li et al. (15)
TC	27	M	Tislelizumab	6	This case

NSCLC, non-small cell lung cancer; GC, gastric cancer; BC, breast cancer; SCLC, small cell lung cancer; TC, Thymic carcinoma; mPSL, methylprednisolone; PSL, prednisolone; M, male; F, female; UN, unknown.

This study had some limitations. Firstly, we did not give timely steroids treatment. Since the patient had a history of kidney stone, our primary thought of the patient’s pain was consequence of kidney stones. When conventional treatment of antibiotics and antispasmodic treatment failed to relive the pain, additional examination of PET/CT and MRU indicated the ureter expansion and cystoscopy indicated cystitis. Based on these results, we considered the pain was caused by tislelizumab-induced ureteritis/cystitis. Secondly, we did not perform cystoscopy after his pain was alleviated because the patient refused. Otherwise, we cloud better observe the changes in the inner wall of the bladder after discontinuing tislelizumab. Thirdly, the diagnosis of cystitis was made based on cystoscopy examination revealing the inflammatory reaction of the inner wall of the bladder, and we did not perform pathological examination.

In conclusion, we reported a rare case of tislelizumab-induced ureteritis/cystitis mainly presented with severe pain and ureteral expansion. This case reminds us of the potential risk of urinary system during ICI treatment. Since tislelizumab is currently used in various malignancies, our case will provide a reference for the diagnosis and treatment of ICI-related irAEs.

## Data availability statement

The original contributions presented in the study are included in the article/**Supplementary Material**. Further inquiries can be directed to the corresponding author.

## Ethics statement

The studies involving humans were approved by Ethics Committee of Zhejiang Provincial People’s Hospital. The studies were conducted in accordance with the local legislation and institutional requirements. The participants provided their written informed consent to participate in this study. Written informed consent was obtained from the individual for the publication of any potentially identifiable images or data included in this article. Written informed consent was obtained from the participant/patient(s) for the publication of this case report.

## Author contributions

QZ designed the case report and drafted the manuscript. ZQ analyzed the patient data and revised the manuscript. PY proposed the concept of this case report. QW and JQ administered the whole course of diagnosis and treatment in this patient. YC analyzed the patient data, and provided significant contributions to the analysis of the patient data. All authors contributed to the article and approved the submitted version.

## Supplementary material

Supplementary Figure 1 Histopathology and immunohistochemistry of the liver biopsy specimens of this patient in April 2022. **(A)** HE staining revealed poorly differentiated carcinomas (20×), IHC staining showed that liver cells were positive for **(B)** CK(Pan) (20×), **(C)** CD5 (20×), **(D)** CD117 (20×), **(E)** GLUT-1 (20×), **(F)** Muc-1 (20×).

Supplementary Figure 2 Cystoscope scan (September 1^st^, 2022).
