# Supplementary figures and images for: Immune-related adverse events with severe pain and ureteral expansion as the main manifestations: a case report of tislelizumab-induced ureteritis/cystitis and review of the literature
Source: Front Immunol. 2023 Oct 6;14:1226993. doi: 10.3389/fimmu.2023.1226993 (PMC10587548; doi:10.3389/fimmu.2023.1226993)

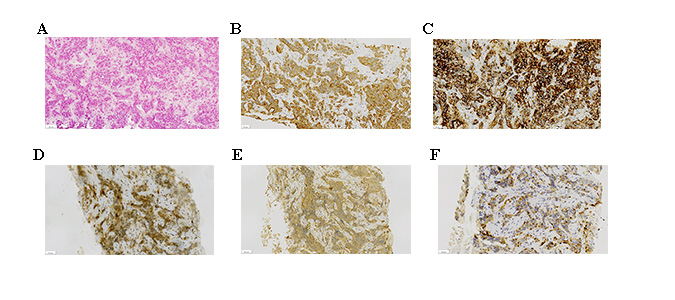

Supplement: Supplementary Figure 1 — Histopathology and immunohistochemistry of the liver biopsy specimens of this patient in April 2022. (A) HE staining revealed poorly differentiated carcinomas (20×), IHC staining showed that liver cells were positive for (B) CK(Pan) (20×), (C) CD5 (20×), (D) CD117 (20×), (E) GLUT-1 (20×), (F) Muc-1 (20×). [file Image_1.jpeg]

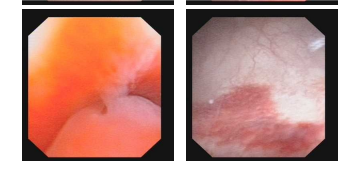

Supplement: Supplementary Figure 2 — Cystoscope scan (September 1st, 2022). [file Image_2.png]
