# Supplementary material for: Immune-related adverse events with severe pain and ureteral expansion as the main manifestations: a case report of tislelizumab-induced ureteritis/cystitis and review of the literature
Source: Front Immunol. 2023 Oct 6;14:1226993. doi: 10.3389/fimmu.2023.1226993 (PMC10587548; doi:10.3389/fimmu.2023.1226993)
Supplement: Supplementary file 3 [file Table_1.docx]

**Table S1. Gene mutation site**

| Gene | Location site | Mutation type | Mutation frequency/number of copies | Mutation grading |
| --- | --- | --- | --- | --- |
| *ALK* | 2:29416087-30143527 | Copy number amplification | 3.3X | 2 |
| *MYCN* | 2:16075981-16090656 | Copy number amplification | 3.0X | 2 |
| *ASXL1* | 20:31022297 | Termination codon obtained mutation | 28.06% | 3 |
| *ASXL2* | 2:25965265 | Missense mutation | 33.46% | 3 |
| *EP300* | 22:41533746 | Termination codon obtained mutation | 35.18% | 3 |
| *NOTCH4* | 6:32190423 | Missense mutation | 38.7% | 3 |
| *PIK3C2B* | 1:204419139 | Missense mutation | 35.43% | 3 |
| *SMAD3* | 15:67473672 | Missense mutation | 17.96% | 3 |
| *SOX2* | 3:181430749 | Missense mutation | 16.96% | 3 |
| *TRAF2* | 9:139794879 | Missense mutation | 44.69% | 3 |
